# Tennessee Delegates to the Medical Convention

**Published:** 1847-05

**Authors:** 


### TENNESSEE DELEGATES TO THE MEDICAL CONVENTION.

The Medical Society of Tennessee have appointed six delegates to the Medical Convention. The names of the delegates are Dr. Hays, of Columbia; Dr. Avent, of Murfreesborough; Drs. Stout, Martin, and Buchanan, of Nashville, and Dr. Nelson, of Davidson county.

